# An Overexpressed *Q* Allele Leads to Increased Spike Density and Improved Processing Quality in Common Wheat (*Triticum aestivum*)

**DOI:** 10.1534/g3.117.300562

**Published:** 2018-01-22

**Authors:** Bin-Jie Xu, Qing Chen, Ting Zheng, Yun-Feng Jiang, Yuan-Yuan Qiao, Zhen-Ru Guo, Yong-Li Cao, Yan Wang, Ya-Zhou Zhang, Lu-Juan Zong, Jing Zhu, Cai-Hong Liu, Qian-Tao Jiang, Xiu-Jin Lan, Jian Ma, Ji-Rui Wang, You-Liang Zheng, Yu-Ming Wei, Peng-Fei Qi

**Affiliations:** Triticeae Research Institute, Sichuan Agricultural University, Chengdu, Sichuan 611130, China

**Keywords:** bread-making quality, compact spike, point mutation, protein content, wheat breeding, mutant screen report

## Abstract

Spike density and processing quality are important traits in modern wheat production and are controlled by multiple gene loci. The associated genes have been intensively studied and new discoveries have been constantly reported during the past few decades. However, no gene playing a significant role in the development of these two traits has been identified. In the current study, a common wheat mutant with extremely compact spikes and good processing quality was isolated and characterized. A new allele (*Q^c1^*) of the *Q* gene (an important domestication gene) responsible for the mutant phenotype was cloned, and the molecular mechanism for the mutant phenotype was studied. Results revealed that *Q^c1^* originated from a point mutation that interferes with the miRNA172-directed cleavage of *Q* transcripts, leading to its overexpression. It also reduces the longitudinal cell size of rachises, resulting in an increased spike density. Furthermore, *Q^c1^* increases the number of vascular bundles, which suggests a higher efficiency in the transportation of assimilates in the spikes of the mutant than that of wild type. This accounts for the improved processing quality. The effects of *Q^c1^* on spike density and wheat processing quality were confirmed by analyzing nine common wheat mutants possessing four different *Q^c^* alleles. These results deepen our understanding of the key roles of *Q* gene, and provide new insights for the potential application of *Q^c^* alleles in wheat quality breeding.

Common wheat (*Triticum aestivum* L.) is one of the most important food crops worldwide, and is important for the establishment of human civilization. Spike density of wheat is an important characteristic that is controlled by multiple loci. The *C* locus on the 2D chromosome of *Triticum aestivum* ssp. *compactum* (Host) Mac Key (club wheat) results in increased spike density (compact spike; Johnson *et al.* 2008). The *Q* gene located on the long arm of chromosome 5A (5AL) affects spike density. *Q* is an AP2 transcription factor, containing two AP2 DNA-binding domains and a miRNA172-binding site in the 10th exon (Simons *et al.* 2006). Four loci (*C^739^*, *C^17648^*, *Cp^m^*, and *Cp*) determining spike density are present in tetraploid wheat and hexaploid wheat as well (Kosuge *et al.* 2008, 2012; Laikova *et al.* 2009; Mitrofanova 1997). It was demonstrated that *C^739^* and *Cp* are different from the well known *Q* and *C* loci (Kosuge *et al.* 2012).

MicroRNAs (miRNAs) are ∼22 nucleotides in length RNAs that repress the expression of genes post-transcriptionally, mainly by DNA elimination, mRNA cleavage, and translational repression (Mallory and Vaucheret 2006). Currently, increasing data demonstrate that miRNAs play critical functions in almost all biological and metabolic processes in plants (Sun 2012). One well studied example is miRNA172, which regulates floral organ identity, flowering time, spike density, and stress response. An accumulation of miRNA172 in *Arabidopsis* results in early flowering, and disrupts floral organ identity (Aukerman and Sakai 2003), with defects in carpels and a reduction in stamen number (Chen 2004; Zhao *et al.* 2007). The regulation between miRNA172 and its targets, *SCHLAFMÜTZE* (*SMZ*) and *SCHNARCHZAPFEN* (*SNZ*) (Schmid *et al.* 2003), represses flowering. Overexpression of miRNA172 in rice causes lower fertility and reduced seed weight (Zhu *et al.* 2009). In barley, the elongation of inflorescence internodes is affected by miRNA172*-HvAP2* regulation, which results in an extreme spike density (Houston *et al.* 2013). Overexpression of *RAP2.1*, which possess the miRNA172 target region, leads to greater sensitivity to cold and drought stress in *Arabidopsis* (Dong and Liu 2010). A transgenic *Arabidopsis* line of soybean miRNA172 shows tolerance to salt stress and increased sensitivity to ABA by regulating its target gene (Li *et al.* 2015). In wheat, a miRNA172-AP2-like system plays a crucial role in regulating of flowering time, and in spike morphogenesis (Debernardi *et al.* 2017). Overexpression of miRNA172 leads to an elongated spike (Debernardi *et al.* 2017; Liu *et al.* 2017).

Processing quality is a valuable trait in wheat breeding and production. Diverse food has been developed to take advantage of the unique properties of wheat flour (*i.e.*, mixing characteristics, dough rheology, and baking performance). Gluten, including high molecular weight glutenin subunit (HMW-GS), low molecular weight glutenin subunit (LMW-GS) and gliadins (Payne 1987), and genes regulating the expression of gluten affect wheat processing quality. *SPA* (Storage Protein Activator), homologous to the *Opaque2* gene in maize, is a key regulator of the expression of gluten in wheat (Ravel *et al.* 2009). A *NA*C (NAM, ATAF, and CUC transcription factor) gene can increase grain protein content (GPC) in wheat (Uauy *et al.* 2006). *DOF* (DNA binding with one finger) can activate the expression of α gliadin genes during grain filling (Dong *et al.* 2007).

Although the genes/gene loci associated with spike density and processing quality have been intensively studied, no gene playing a significant role in the development of these two traits was identified. In the current study, a common wheat mutant, with increased spike density and improved processing quality, was isolated. We demonstrated that a point mutation within the miRNA172-binding site of *Q* gene altered its transcriptional level, which is responsible for the mutant phenotype. This research deepens our understanding of the *Q* gene in wheat development, and provides new insights for the potential utilization of the *Q* gene in wheat.

## Materials and Methods

### Wheat materials and growth conditions

A common wheat mutant (*S-Cp1-1*) with increased spike density was isolated from 0.6% ethyl mesylate (EMS)-treated common wheat (*Triticum aestivum* L.) cultivar “Shumai482.” *S-Cp1-1* and its corresponding wild type (WT) used were isolated from an M_6_ heterozygous plant. Nine independent mutants related to *S-Cp1-1* (Supplemental Material, Table S1 in File S1) were obtained from 0.8% EMS-treated *T. aestivum* cv “Shumai482,” “Liangmai4,” “Mianmai37,” and “Roblin,” respectively. *S-Cp1-1* was used as the female parent in crossing with br220 (a hexaploid wheat line) and wanke421 (a common wheat cultivar), to construct segregation populations. The plants were grown at the experimental farm of Sichuan Agricultural University in Wenjiang, with row spacing of 20 *×* 10 cm. A nitrogen: phosphorous: potassium (15: 15: 15; 450 kg per hectare) compound fertilizer was used before sowing.

To assess the effect of mutant gene on processing quality, *S-Cp1-1* and its WT were grown at the experimental farm in Wenjiang as well, in a randomized block design with three replicates for two growing seasons (2014–2015 and 2015–2016). Each replicate was planted with an area of 2 × 4 m, with row spacing of 20 *×* 5 cm. A nitrogen: phosphorous: potassium (15: 15: 15; 450 kg per hectare) compound fertilizer was used before sowing.

### Microscopy analysis

The developing spikes of *S-Cp1-1* and WT were scanned using an optical microscope (Olympus, Tokyo, Japan) and EPSON perfection V700 (EPSON, Tokyo, Japan). The spikes at GS59 (decimal code of wheat development; Zadoks *et al.* 1974) were fixed in FAA solution (70% alcohol: 37% formaldehyde: acetic acid = 18: 1: 1, v: v: v) and embedded in paraffin. Then, the paraffin wax was cut into 6-μm sections using a Leica slicer (Leica, Wetzlar, Germany). Safranin O/fast green (Solarbio, Beijing, China) was used for staining. The splices were photographed by using a BX60 light microscope (Olympus).

### Genetic mapping and gene cloning

Genomic DNA was extracted from the young leaves of F_2_ individuals derived from *S-Cp1-1 ×* br220 (Doyle and Doyle 1987). Genomic DNAs of 10 randomly selected F_2_ individuals with compact spike/normal spike were pooled, and 24 DNA pools were constructed. The pooled DNA samples were analyzed by Illumina 90K single nucleotide polymorphism (SNP) microarray at Compass Biotechnology (Beijing, China), to primarily locate the mutant locus. The locus was then mapped by sequence-tagged site (STS) and simple sequence repeat (SSR) markers in an F_2_ population containing 819 plants, derived from *S-Cp1-1 ×* br220. STS and SSR markers (Table S2 in File S1) were developed based on the SNP markers and the physical map draft of *Triticum urartu* (Ling *et al.* 2013; http://plants.ensemble.org/index.html; http://www.gramene.org/gremene/searches/ssrtool). The genetic linkage map was constructed according to the method described by Jiang *et al.* (2014).

The cDNA and genomic DNA sequences of candidategene were PCR amplified from both mutant and WT plants, and confirmed by Sanger sequencing (Invitrogen, Shanghai, China). Sequences were analyzed by DNAMAN V6 (Lynnon Biosoft, San Ramon). The primers used are listed in Table S2 in File S1.

### RNA extraction and qRT-PCR analysis

The spikes of *S-Cp1-1* and its WT counterpart were collected at GS24, GS29, and GS32 (Zadoks *et al.* 1974). There were three biological replicates for each stage, with at least 10 spikes for each replicate. Root, stem, and leaf samples of *S-Cp1-1* and its WT at GS24 stage were collected as well. Three biological replicates were done for each tissue, with 10 plants used per replicate. The harvested samples were ground in liquid nitrogen, and RNA was extracted using the Plant RNA extraction kit V1.5 (Biofit, Chengdu, China).

qRT-PCR reactions were performed using a SYBR premix Ex Taq RT-PCR kit (Takara, Dalian, China). RNA clean up, cDNA synthesis, qRT-PCR analyses were performed as described in Wang *et al.* (2010). Two housekeeping genes (Long *et al.* 2010), *i.e.*, Scaffold-associated regions (SAR) DNA binding protein (NCBI UniGene Ta.14126) and methionine aminopeptidase 1 (Ta.7894), were amplified as reference genes for normalization of data. The primers used for qRT-PCR are listed in Table S2 in File S1.

### 5′ modified RACE

The 5′ rapid amplification of cDNA end (RACE) analysis was performed as in Llave *et al.* (2002). Total RNA was extracted from spikes of *S-Cp1-1* and its WT at the GS24 using a Plant RNA extraction kit V1.5 (Biofit). The primers for the first and second PCR products were Q-cDNA-R and 3′RACE-R (Table S2 in File S1), respectively. The second PCR products were purified and cloned into the pMD19-T vector (Takara) for sequencing.

### SDS-PAGE analysis

Seed storage proteins were extracted from 20 mg whole wheat flour and separated by sodium dodecyl sulfate-polyacrylamide gel electrophoresis (SDS-PAGE) as described by Qi *et al.* (2011).

### Processing quality analysis

Grain samples were cleaned and adjusted to 14% moisture, before being milled with a Brabender Quandrumat Junior mill (Brabender, Duisburg, Germany). The grain protein content (dry weight), zeleny sedimentation value and wet gluten content were measured following GB/T 17320-2013, using an automatic azotometer (Kjelec 8400; FOSS, ‎Hillerød, Denmark), a zeleny analysis system (CAU-B, Beijing, China), and a glutomatic 2200 system (Perten, Hägersten, Sweden), respectively.

Dough rheological properties were determined with a 10-g Mixograph (TMCO, Lincoln, NE). Samples were mixed to optimum water absorption following the 54-40A method (AACC 2001). The development time (minutes to the curve peak) was measured. Finally, results were collected and analyzed using the MixSmart software.

The baking test was performed according to AACC method 10.09-01 (AACC 2010) with some modifications. The baking procedure was the standard rapid-mix-test with 40 g flour at 14% moisture content. Three biological replicates were conducted with two breads for each flour sample. The loaf volume was determined by BVM6630 volume meter (Pertern) following the manufacturer’s instruction.

### Statistical analysis

The chi square (χ*^2^*) test (for mutant phenotype), *t*-test (for processing quality), and least-significant difference (LSD) test under a general linear model (for processing quality) were carried out using DPS software (version 12.01; Tang and Zhang 2013).

### Data availability

All data necessary for the conclusions are represented in the paper’s tables, figures and supplemental information. The mutants are available upon request. Nucleotide sequence data from this article can be found in the GenBank database under the following accession numbers: KX580301–KX580304 and KX620761–KX620768.

## Results

### Identification of new Q alleles

**Figure 1:**
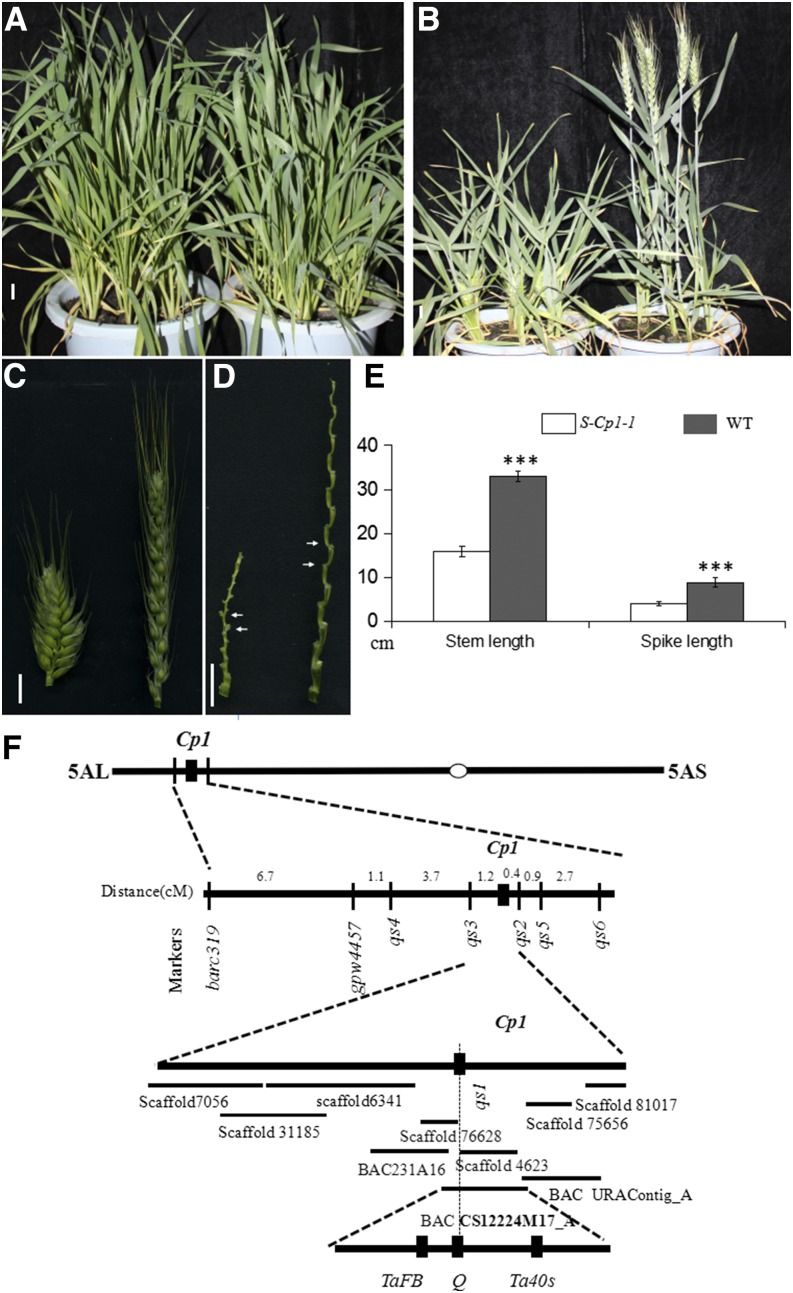
Phenotype of *S-Cp1-1* and mapping of the *Cp1* locus. (A) Plants of *S-Cp1-1* (left) and WT (right) at GS29. (B) Plants of *S-Cp1-1* (left) and WT (right) at GS59. (C) Spikes of *S-Cp1-1* (left) and WT (right) at GS59 (D) Rachises of *S-Cp1-1* (left) and WT (right) at GS59. The rachises between white arrows were used in Figure 6. (E) Stem and spike lengths of *S-Cp1-1* and WT at GS90. Data are means ± SD (SD; *n* = 35). *** *P* < 0.01. (F) Mapping of the *Cp1* locus. The BACs (bacterial artificial chromosomes) and genomic scaffolds were queried using a BLASTN algorithm in NCBI (http://www.ncbi.nlm.nih.gov/) and aligned based on their relative positions and overlap. All of the BACs and scaffolds used are listed in Table S3 in File S1. Scale bar (A–D), 1 cm.

**Figure 2:**
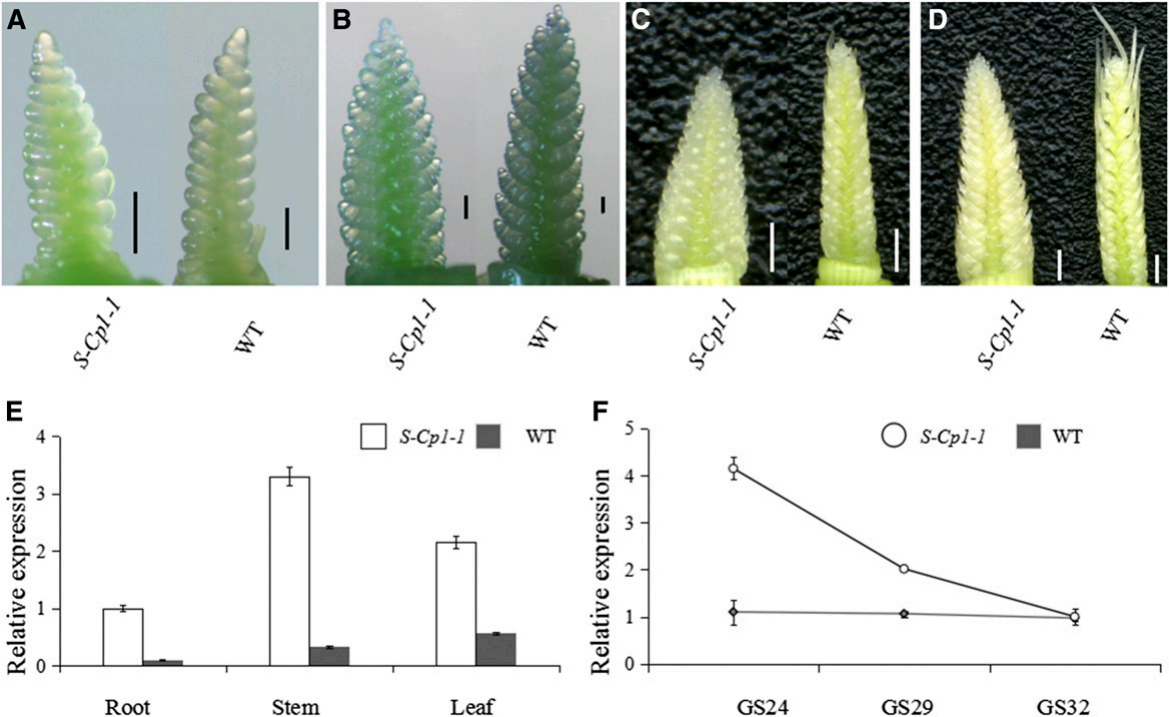
Expression of *Q^c1^* measured by qRT-PCR. (A–D) Spikes of *S-Cp1-1* (left) and WT (right) at GS22, GS24, GS29, and GS32, respectively. Scale bar, 0.1 cm (A and B) and 1 cm (C and D). (E) Relative expression levels of *Q^c1^* and *Q* in root, stem, and leaf at GS24. (F) Relative expression of *Q^c1^* and *Q* at GS24, GS29, and GS32. Error bars represent means ± SD (*n* = 3).

A common wheat mutant (*S-Cp1-1*), with increased spike density (compact spike) and improved processing quality (Figure 1 and Table 1), was isolated. *S-Cp1-1* had a similar architecture to its WT before GS30 (decimal code of wheat development; Zadoks *et al.* 1974; Figure 1A). Thereafter, its plant height was gradually lower than that of the WT (Figure 1B). The spike density of *S-Cp1-1* differed from that of WT earlier than GS22 (Figure 2A). The increased spike density and reduced plant height were not separated in the BC_1_F_2_ population (1010 individuals), suggesting that they are controlled by the same locus. *S-Cp1-1* was used as the female parent in a cross with br220 (a hexaploid wheat line), to develop a mapping population. To facilitate genetic research, the increased spike density was selected as the target trait for mapping. A chi square (χ*^2^*) test showed that the spike density of 819 F_2_ plants derived from *S-Cp1-1* × br220 matched a theoretical 3:1 segregation ratio (*χ*^2^ = 0.12; *P* = 0.69), suggesting that the increased spike density in *S-Cp1-1* was controlled by a single dominant locus (*Cp1*).

**Table 1:** Comparison of processing quality parameters of *S-Cp1-1* to its WT

	GPC (%; Dry Weight)		WGC (%)		Zeleny Sedimentation Value (ml)		Development Time (min)	
	2014–2015	2015–2016	2014–2015	2015–2016	2014–2015	2015–2016	2014–2015	2015–2016
*S-Cp1-1*	19.72A	17.90A	50.60A	41.95A	63.05A	45.87A	7.11A	3.23A
WT	14.00B	11.49B	34.83B	20.09B	36.83B	20.09B	2.46B	1.41B
	*F* value	*P* value	*F* value	*P* value	*F* value	*P* value	*F* value	*P* value
E	242.28	<0.01	221.15	<0.01	51.67	<0.01	295.41	<0.01
G	1909.72	<0.01	574.10	<0.01	110.61	<0.01	360.68	<0.01
G × E	5.96	0.041	15.12	<0.01	0.73	0.417	55.27	<0.01

The seeds were harvested in two growing seasons, *i.e.*, 2014–2015 and 2015–2016. “A” and “B” represent significance at *P* < 0.01. Significance was calculated by using *t*-test and LSD test. E, environment; G, genotype.

**Figure 3:**
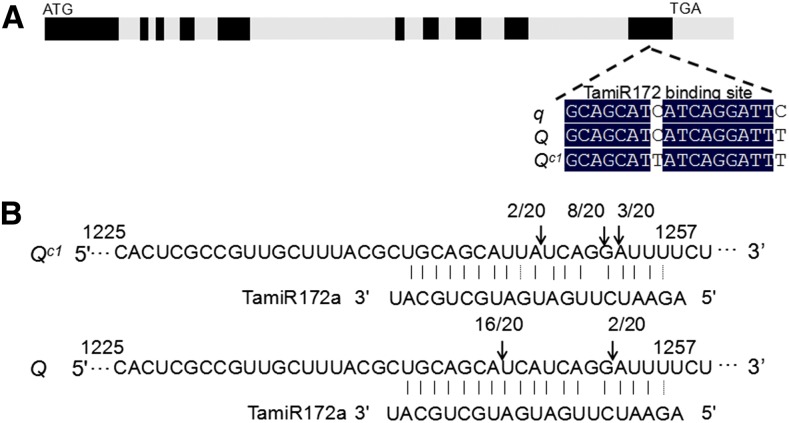
Genomic structure of *Q^c1^* and confirmation of miRNA172-directed regulation in the developing spike at GS24. (A) Genomic structure of the *Q^c1^*. The initiation and termination codons, exons (black rectangles), and introns (gray rectangles) are illustrated. The point mutations in the miRNA172-binding site of *q*, *Q*, and *Q^c1^* are indicated. (B) miRNA172 cleavage sites in the transcripts of *Q^c1^* and *Q* as determined by 5′ RACE.

*Cp1* was positioned on 5AL (Figure S1 in File S1, Table S4), by using a wheat 90K SNP microarray and 24 DNA pools of F_2_ plants generated from *S-Cp1-1* × br220. Subsequently, STS and SSR markers (Table S2 in File S1) were developed. *Cp1* was placed in a 1.6 cM region between markers *qs2* and *qs3*, with 0.4 and 1.2 cM in the F_2_ population, respectively Table S5. Notably, the chromosome region flanked by *qs2* and *qs3* contained the *Q* gene, whose overexpression increases spike density (Simons *et al.* 2006). *Q* is an AP_2_ transcription factor, containing a miRNA172-binding site in the 10th exon. To demonstrate whether the increased spike density in *S-Cp1-1* was caused by *Q*, we developed an intragenic molecular marker (*qs1*) for *Q* gene. *qs1* cosegregated with compact spike in 10,100 F3 individuals derived from *S-Cp1-1* × br220. One missense mutation (C-T; serine to leucine; Figure 3A and Figure S2 in File S1) in the miRNA172-binding site of *Q* was identified (GenBank nos. KX580301 and KX580302). To facilitate the following description, we named the new *Q* allele as *Q^c1^* (the first *Q* leading to compact spike).

**Figure 4:**
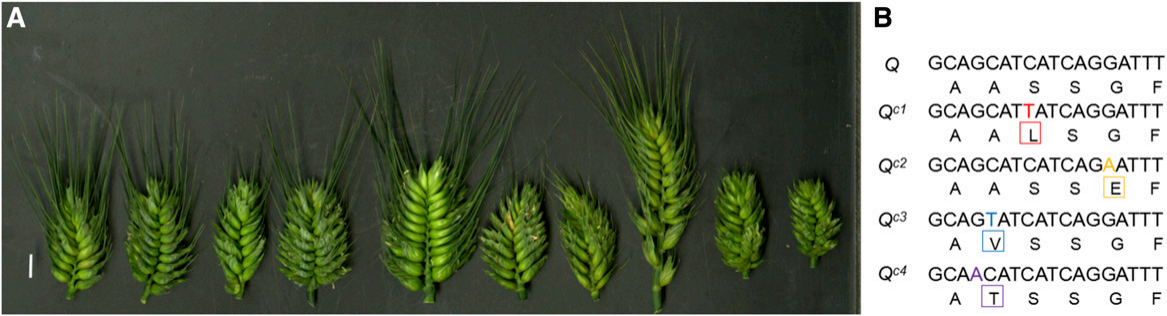
Molecular characterization of the four *Q^c^* alleles. (A) Features of the spikes of *S-Cp1-1*, *S-Cp1-2*, *R-Cp1-3*, *L-Cp2-1*, *M-Cp2-2*, *R-Cp2-3*, *R-Cp2-4*, *S-Cp3-1*, *R-Cp3-2*, and *R-Cp4-1* at GS70 (left to right; Table S1 in File S1). (B) Polymorphisms of the four *Q^c^* alleles and their predicted amino acid substitutions.

The effect of *Q^c1^* on spike density was confirmed by analyzing nine independent common wheat mutants possessing four different *Q^c^* alleles (*Q^c1^-Q^c4^*; Figure 4 and Table S1 in File S1). These alleles contained four different point mutations in the miRNA172-binding site, supporting a causal relationship between the transcriptional regulation of *Q* by miRNA172 and the mutant phenotype.

### Comparison of expression levels

The transcriptional levels of *Q^c1^* and *Q* were compared by qRT-PCR. The ratios of transcriptional level of *Q^c1^* to that of *Q* were 3.8 at GS24 (Figure 2, B and F), 1.9 at GS29 (Figure 2, C and F), and one at GS32 (Figure 2, D and F), respectively. It revealed that the increased spike density in *S-Cp1-1* was a result of higher transcriptional level of *Q^c1^* in developing spike before GS32 (Figure 2, A–D). Besides spike, *Q^c1^* and *Q* expressed differentially at the RNA level in root, stem, and leaf at GS24 as well. Relative to *Q*, the transcriptional levels of *Q^c1^* were 10.2-fold in root, 9.9-fold in stem, and 3.8-fold in leaf (Figure 2E).

To validate whether the higher transcriptional level of *Q^c1^* was due to altered cleavage directed by miRNA172, 5′ RACE analysis was carried out. The sequencing of 20 randomly chosen *Q^c1^* and *Q* clones showed that the cleavage site in the miRNA172-binding region was changed. We can conclude that the point mutation in *Q^c1^* disturbed *in vivo* cleavage by miRNA172 (Figure 3B), suggesting that the phenotype of *S-Cp1-1* was due to overexpression of *Q*, resulting from the point mutation that interferes with the miRNA172-directed cleavage of the *Q* transcripts.

### Effect of new Q alleles on grain quality

**Figure 5:**
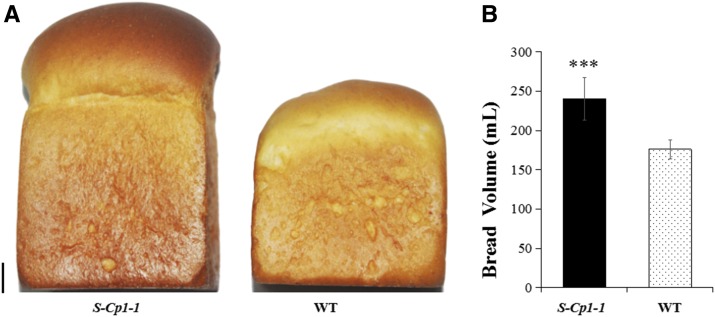
*Q^c1^* increases loaf volume. (A) Intact loaves of *S-Cp1-1* and its WT. Scale bar, 1 cm. (B) Comparison of loaf volume of *S-Cp1-1* to its WT. “***” above column indicates the significance at *P* < 0.01.

Four parameters reflecting wheat processing quality were compared between *S-Cp1-1* and its WT in two growing seasons (Table 1). In contrast to the WT control, GPC, wet gluten content, zeleny sedimentation value, and development time were significantly higher (*P* < 0.01) for *S-Cp1-1*. The average of loaf volume of *S-Cp1-1* was 37% greater (*P* < 0.01) than that of the WT (Figure 5). No variation in the composition of gluten was observed between *S-Cp1-1* and WT (Figure S3 in File S1), especially HMW-GS, which is among the most important determinants in bread-making quality (Shewry *et al.* 2003). To assess the effect of *Q^c1^* on processing quality in different genetic backgrounds, GPC of individual plants belonging to two F_2_ populations was measured (Table 2). As expected, GPC of plants with two copies of *Q^c1^* allele was significantly higher than those with one or no *Q^c1^* copies (*P* < 0.01). The effect of *Q^c1^* on processing quality was confirmed by analyzing independent common wheat mutants possessing four different *Q^c^* alleles (Table S1 in File S1) as well.

**Table 2:** Effect of *Q^c1^* on grain protein content (dry weight) in two F_2_ populations

	GPC (%)	
	*S-Cp1-1* × Br220	*S-Cp1-1* × wanke421
*Q^c1^*/*Q^c1^*	20.00A	22.43A
*Q^c1^*/*Q*	14.56B	17.19B
*Q*/*Q*	11.88C	10.32C
	*F* value	*P* value
Population	46.1	<0.01
Genotype	1163.0	<0.01
P × G	63.6	<0.01

The seeds of 20 individual F_2_ plants were harvested for each of the lines with zero, one or two *Q^c1^* copies. “A,” “B” and “C” indicate significance at *P* < 0.01. Significance was calculated by using *t*-test and LSD test.

### Effect of Q^c1^ on cells of rachis

**Figure 6:**
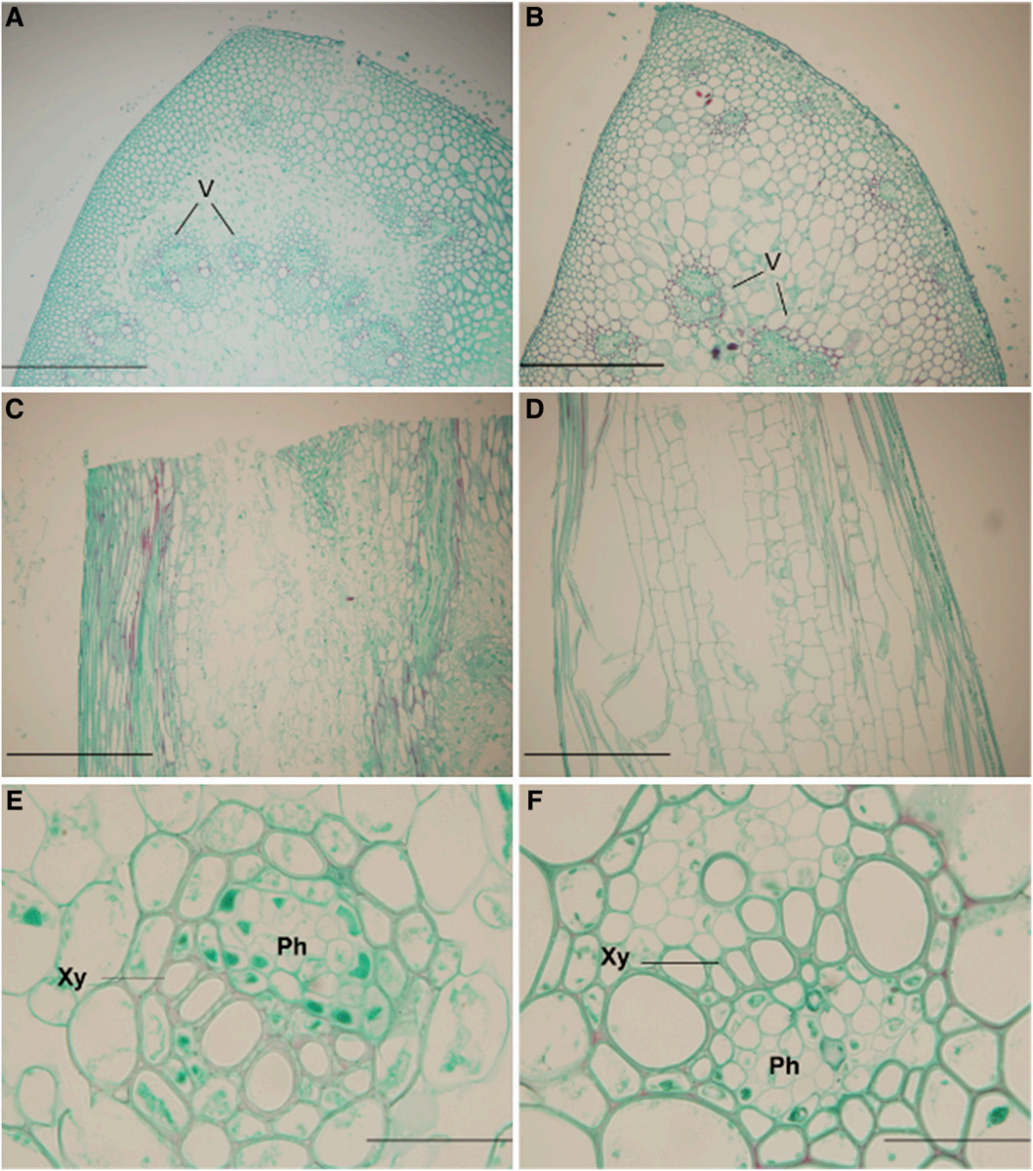
Contrasting cell morphology of the rachises of *S-Cp1-1* and its WT at GS59. (A and B) The transverse sections of *S-Cp1-1* (A) and WT (B). (C and D) The longitudinal sections of *S-Cp-1* (C) and WT (D). (E and F) The cells in the vascular bundles of *S-Cp1-1* (E) and WT (F). V, vascular bundles; Ph, phloem; Xy, xylem. Scale bars, 10 μm (A–D) and 0.1 μm (E and F).

A microscopic comparison of the longitudinal sections of rachis indicated that the cells in *S-Cp1-1* were decreased in size (Figure 6C) compared with those in the WT (Figure 6D). It is obvious that *Q^c1^* reduced the longitudinal cell size of rachis, resulting in increased spike density in *S-Cp1-1*. Transverse sections of rachis revealed that cells of *S-Cp1-1* were reduced in size and increased in number, and, notably, the number of vascular bundles in *S-Cp1-1* was increased (Figure 6, A and B). The increase in the number of vascular bundles suggested a higher efficiency in the transportation of assimilates in the spikes of the mutant than that of WT. This accounts for the improved processing quality of *S-Cp1-1*. Additionally, the vascular morphology was changed in *S-Cp1-1* (Figure 6A). There were a lower number of xylem cells in the vascular bundles, and a greater number of cells around the vessels (Figure 6, A and E) when compared with the WT (Figure 6, B and F).

## Discussion

During the domestication of common wheat, changes in gross morphology of the spike enhanced its suitability for wheat production. There are three major genes that affect gross morphology of the spike in common wheat, *i.e.*, *Q*, *C*, and *S1* (Morris and Sears 1967), which have taxonomic importance. The *Q* gene on chromosome 5AL pleiotropically influences many characters, including spike density and seed threshability (Faris and Gill 2002; Simons *et al.* 2006). The *C* gene on chromosome 2D (Johnson *et al.* 2008) genetically controls the compact spike in a subspecies of hexaploid wheat known as *T. aestivum* ssp. *compactum*. The *S1* gene on chromosome 3D determines the unique spike morphology of *T. aestivum* ssp. *sphaerococcum* (Sears 1947). The results of the current study indicate an effect on spike density similar to that the *C* gene of a new *Q* allele, suggesting that there is some similarity between the molecular pathways of these two genes in regulating spike density.

As a major domestication gene in wheat, *Q* arose through a point mutation occurring in the miRNA172-binding site of *q* (Figure 3A; Simons *et al.* 2006). *Q^c^* alleles originated from the introduction of more point mutations into the miRNA172-binding site of *Q* (Figure 4B). Interestingly, the transcriptional levels of *q*, *Q*, and *Q^c1^* are correlated with the number of point mutations in the miRNA172-binding site, indicating that post-transcriptional regulation plays a critical role in the expression of the *Q* gene (Figure 2, E and F; Simons *et al.* 2006). Greenwood *et al.* (2017) reported a relationship between a point mutation in the miRNA172-binding site of *Q* (equivalent to the *Q^c2^* allele in this paper) and spike density. Here, we identified three new point mutations in the miRNA172-binding site of *Q* in different genetic backgrounds (Figure 4), further demonstrating that overexpression of *Q* is the causal mechanism for the observed change in spike morphology in mutants.

For miRNA-directed cleavage, base-pairing between miRNAs and their target mRNAs is critical (Huntzinger and Izaurralde 2011). The most important feature for mRNA–miRNA pairing is the “seed site” (Bartel 2009), which is 2–7 nt from the 5′ region of miRNAs. The point mutations in *q* and *Q*’ (*Q^c2^* allele in this paper) (Simons *et al.* 2006; Greenwood *et al.* 2017) occur in the seed site, which induce dramatic phenotype changes. However, what happens if the point mutations occur outside the seed site of miRNA172 binding region of *Q* gene remains unclear. It was uncertain whether point mutations outside the seed site interfere with the cleavage of *Q* and ultimately bring about kindred or a new phenotype. We identified three new alleles (*Q^c1^*, *Q^c3^*, and *Q^c4^*) in different genetic backgrounds with nucleotide polymorphisms outside the seed site of the miRNA172 binding region of the *Q* gene. The mutants with these new alleles exhibit similar phenotypes as that of *Q^c2^* (Figure 4 and Table S1 in File S1), indicating that the seed site of the miRNA172 binding region within *Q* gene is not as strict as expected.

Consistent with the results of Liu *et al.* (2017) and Greenwood *et al.* (2017), our 5′ RACE analysis show that the point mutation in the miRNA172-binding site can disturb *in vivo* cleavage by miRNA172, leading to overexpression of the *Q* gene. Furthermore, inhibition of miRNA172 activity by a miRNA target mimic resulted in compact spikes (Debernardi *et al.* 2017). Overexpression of bread wheat miRNA172 caused a speltoid-like spike phenotype (Liu *et al.* 2017). These results point to a critical role of miRNA172 in regulation of the *Q* gene at the transcript level.

Improvements in wheat processing quality have been studied extensively over the years. However, the effect of *Q* on wheat processing quality was rarely studied. The unique properties of wheat flour depend primarily on seed storage proteins—one of the most important sources of protein for human beings—which consist mainly of glutenins and gliadins (Payne 1987; Shewry *et al.* 2003). These latter proteins are responsible for dough elasticity and extensibility. Diverse food has been developed to take advantage of the properties of wheat flour. Despite its significance in human life, efforts to improve the processing quality of wheat have been hindered by a complex genetic system and strong environmental effects (Simmonds 1995). Wheat processing quality is a complex of characteristics controlled by a large number of genes (Ma *et al.* 2009). GPC is a crucial index for measuring wheat quality (Weegels *et al.* 1996), and is a frequent target in wheat breeding. The genetic components of GPC in wheat have been extensively studied for many years. The greatest effect was detected by Joppa *et al.* (1997), who found a QTL explaining 66% of the phenotypic variation of GPC. The identified gene in this QTL encodes a NAC transcription factor, which is associated with a GPC increases of ∼14 g kg^−1^ (Uauy *et al.* 2006). In contrast to *Q*, *Q^c1^* is correlated with GPC increases of ∼60 g kg^−1^ (Table 1), suggesting a key role of *Q* in regulating the accumulation of seed storage proteins in wheat. It is well known that *Q* has a profound effect on the spread of polyploid wheat, since, in contrast to the *q* allele, it allowed early farmers to easily harvest wheat. Considering the significant effect of *Q^c1^* on GPC and loaf volume (Figure 5 and Table 1), we can speculate that processing quality and nutritional quality might have been important factors for selection of *Q* by early farmers as well. It will be interesting to compare GPC of wheat near isogenic lines for *q*, *Q*, and *Q^c^* alleles.

Compared to *Q*, the *Q^c1^* allele reduces the longitudinal cell size of rachises, resulting in an increased spike density, which is not a favorable character in most wheat-growing areas. Consistent with the results of Simons *et al.* (2006), Greenwood *et al.* (2017) indicated that amino acid replacement in the AP_2_ domain of Q can decrease spike density. Therefore, it is very hopeful that we will be able to obtain wheat lines with new *Q* alleles that contribute to processing quality improvement without affecting spike morphology. Liu *et al.* (2017) suggested the potential role of the bread wheat transcriptional corepressor TOPLESS (TaTPL) in the regulation of spike density. The N-terminal ethylene-responsive element binding factor-associated amphiphilic repression (EAR) (LDLNVE) motif mediates interaction of Q protein with TaTPL. Jost *et al.* (2016) demonstrated the effect of a homolog of Blade-On-Petiole 1 and 2 (BOP1/2) on internode length and homeotic changes of the barley inflorescence. Determination of the interaction between *Q* and the known and unknown genes would be helpful to understand the molecular mechanisms on spike density, and thus be helpful to promote the utilization of *Q^c^* alleles in wheat breeding.

In summary, we characterized a new allele for the *Q* gene—an important domestication gene—and demonstrated that point mutations in the miRNA172-binding site altered the transcriptional level of *Q* gene during the development of wheat spike, which contributes to increased spike density and improved processing quality of mutants. These results deepen our understanding of the key roles of the *Q* gene, and provide new insights for the potential application of *Q^c^* alleles in wheat quality breeding.

## Supplementary Material

Supplemental material is available online at www.g3journal.org/lookup/suppl/doi:10.1534/g3.117.300562/-/DC1.
