# Supplementary material for: An Overexpressed Q Allele Leads to Increased Spike Density and Improved Processing Quality in Common Wheat (Triticum aestivum)
Source: G3 (Bethesda). 2018 Jan 22;8(3):771–8. doi: 10.1534/g3.117.300562 (PMC5844298; doi:10.1534/g3.117.300562)
Supplement: Supplementary file 3 [file 771FileS1.doc]

**
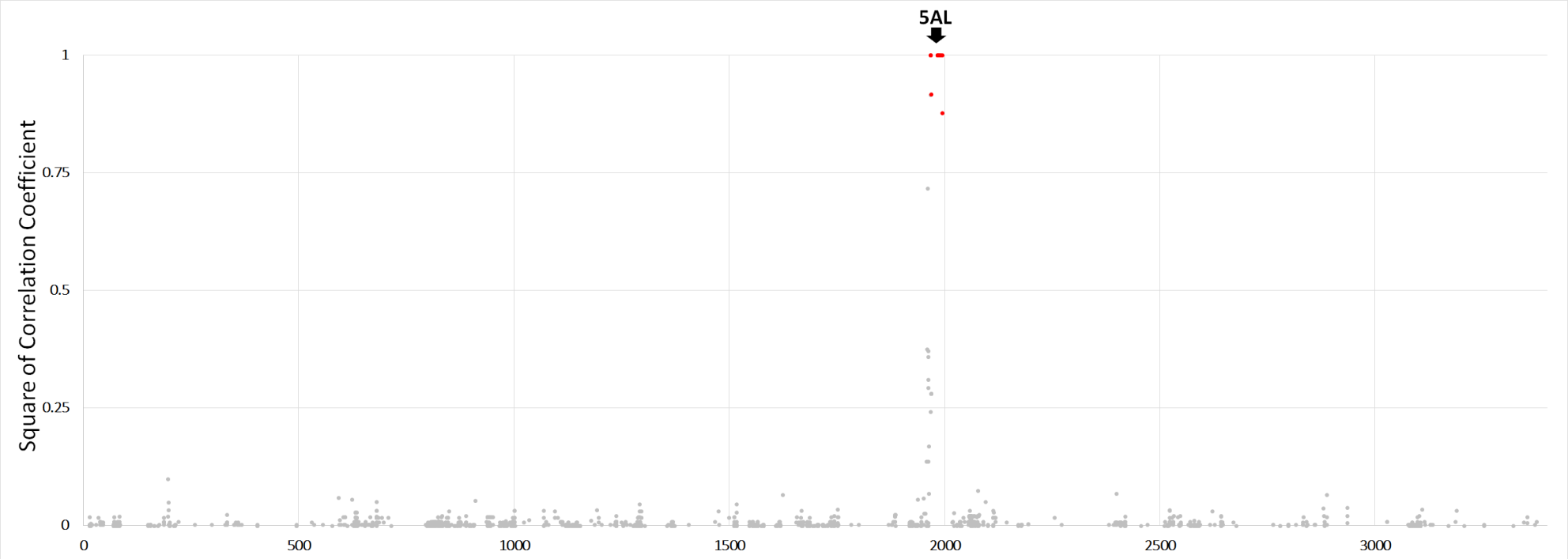
**

**Figure S1** Mapping of the *Cp1* locus by using SNP markers. Dots indicate the markers. The vertical axis shows the square of correlation coefficient between SNP maker and compact spike.


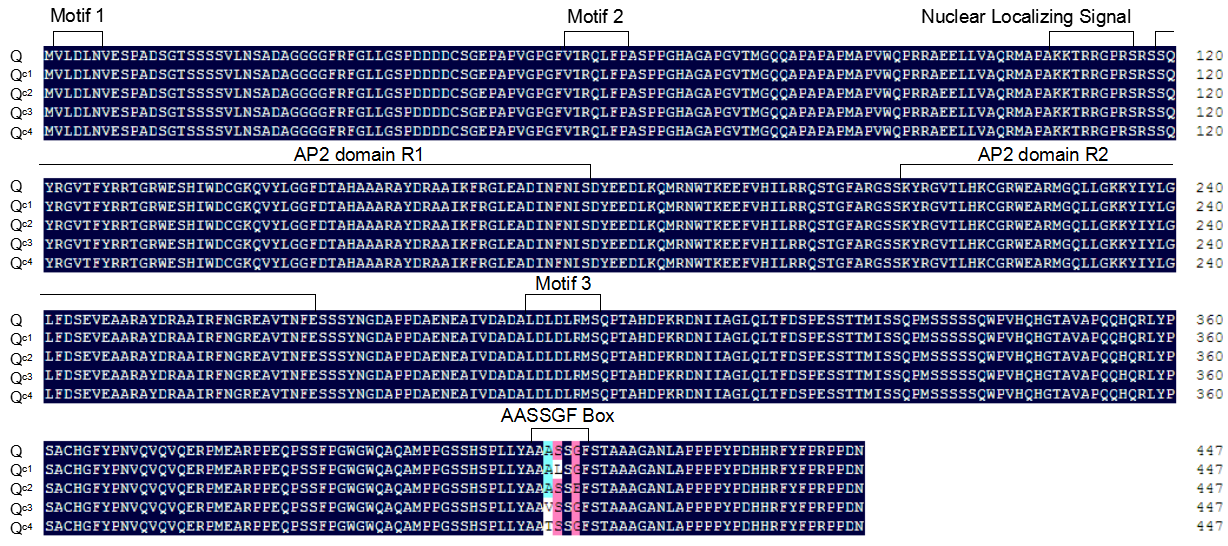


**Figure S2** Alignment of the deduced amino acid sequences of Q and four *Q^c^* alleles. Distribution pattern of conserved domains was taken from Gil-Humanes *et al*. (2009).


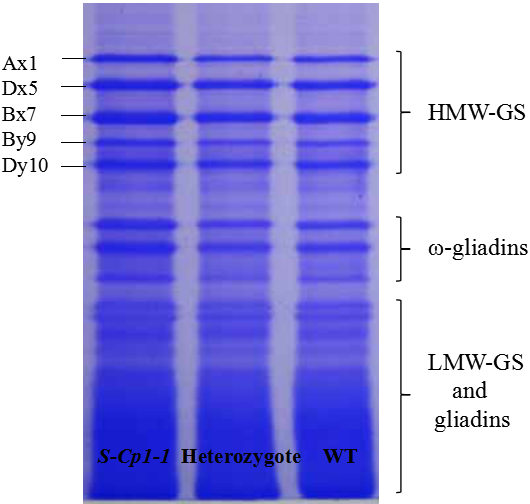
b

**Figure S3** Separation of seed storage proteins by SDS-PAGE. HMW-GS, high molecular weight glutenin subunit; LMW-GS, low molecular weight glutenin subunit; Ax1, Dx5, Bx7, By9 and Dy10 indicate the composition of HMW-GS

**Table S1** Effect of four *Q^c^* alleles on grain protein content (dry weight) in different genetic backgrounds.

| Allele | Mutant | Source | Generation | Grain protein content (%) |
| --- | --- | --- | --- | --- |
| *Q^c1^* | *S-Cp1-2* | Shumai482 | M_5_ | 21.63* (WT 14.75) |
|  | *R-Cp1-3* | Roblin | M_3_ |  |
| *Q^c2^* | *L-Cp2-1* | Liangmai4 | M_5_ | 23.15* (WT 15.63) |
|  | *M-Cp2-2* | Mianmai37 | M_5_ | 22.23* (WT 14.78) |
|  | *R-Cp2-3* | Roblin | M_3_ |  |
|  | *R-Cp2-4* | Roblin | M_3_ |  |
| *Q^c3^* | *S-Cp3-1* | Shumai482 | M_5_ | 19.02* (WT 12.07) |
|  | *R-Cp3-2* | Roblin | M_3_ |  |
| *Q^c4^* | *R-Cp4-1* | Roblin | M_3_ | 22.12* (WT 10.66) |

Wheat lines in the M_3_ generation were not used for measurements, except for *R-Cp4-1*, to ascertain the uniformity of genetic background. All the mutants and their WT (wild type) lines were derived from a single plant in the M_4_ or M_2_ generation. “*” indicates the significance at *P* < 0.01.

**Table S2** Primers used in this study.

| Name | Sequence (5′-3′) | Reference |
| --- | --- | --- |
| Primers of PCR-based molecular markers developed in the study | |  |
| qs1-F | TGAAGCAGGTAATCTTATCCA | This study |
| qs1-R | TGATGACCCTACCCTAAACC | This study |
| qs2-F | CCTCCCTTGATTTCCATT | This study |
| qs2-R | TTCACCTCTAGCCTACCC | This study |
| qs3-F | GTGTTGGTCTACGGATTT | This study |
| qs3-R | GGAAGAAGCGTATTGTGA | This study |
| qs4-F | ACAACTCATACATGGCTTTA | This study |
| qs4-R | ATAGGCCGTGCTACTTTA | This study |
| qs5-F | TCGCCTTCAGTCTTCTCG | This study |
| qs5-R | TCTTGCCTCCTTGGGTTT | This study |
| qs6-F | ACCCGCTATGGCACAATG | This study |
| qs6-R | AGCTGGGCTCCAACCTTT | This study |
| Xgpw4457F | GGTGGTTAGCCGACACATTT | Jiang *et al*. 2014 |
| Xgpw4457R | GCAGATTGATTGCGTCTGG | Jiang *et al*. 2014 |
| Barc319F | GCAGAGCTACGGCAATGT | Kosuge *et al*. 2012 |
| Barc319R | GCGTAAGTCCGGGAAGTAACAGAA | Kosuge *et al*. 2012 |
| Primers used to clone the gene sequence of *Q* | |  |
| AP2startF | ATGGTGCTGGATCTCAATGTGGAGTCGCCGGCGGA | Simons *et al*. 2006 |
| AP2.8R | CGCGGCCAAATCGGGGCAAAGGAATTCAAACGA | Simons *et al*. 2006 |
| WAP2.2F | CACTGGATAATTTCTTCAGGTGGTTTCGACACTGC | Simons *et al*. 2006 |
| AP2.15R | ACATGGAACCTTAATTTCAGGAACGAACTTGTCG | Simons *et al*. 2006 |
| AP2.16F | CTGCTTGGTGCGCTGCTCCACCAGCTTACTGAAA | Simons *et al*. 2006 |
| AP45.1R | CAGAAGGCCCAACGGTTAACGCAACAATGGC | Simons *et al*. 2006 |
| Q-cDNA-F | ATGCCATAGACGCGACCCCA | This study |
| Q-cDNA-R | CCGCCGTCTGGTCACAAC | This study |
| Primers used for 5’ RACE, realtime PCR and reverse transcription PCR | |  |
| qmF3 | ATGGCACGGCAGTAGCACCTCA | This study |
| qmR2 | CAGCGTAAAGCAACGGCGAGTG | This study |
| 3’race R | GGGAAGTAGAACCGGTGGTG | This study |
| Ta.14126.1-F1 | GAGTCTGCCCACCCATTCGTAA | Long *et al*. 2010 |
| Ta.14126.1-R1 | GACATGCCATAGGTTTCAGCGAC | Long *et al*. 2010 |
| Ta.7894.2-F2 | AGCAAGTTGTGACCCGAGGA | Long *et al*. 2010 |
| Ta.7894.2-R1 | GGCGTCAGCAAATAGCAAGTG | Long *et al*. 2010 |

**Table S3** Scaffolds and BACs used in this study.

| Genbank No. | Description | Reference |
| --- | --- | --- |
| JF701616 | *Triticum urartu* clone BAC URAContig_A | Zhang *et al*. (2011) |
| JF701619 | *Triticum aestivum* cultivar Chinese Spring clone BAC CS12224M17_A | Zhang *et al*. (2011) |
| JF701920 | *Triticum turgidum* subsp. *durum* clone BAC LDN376H15_A | Zhang *et al*. (2011) |
| KD284699 | *Triticum urartu* cultivar G1812 unplaced genomic scaffold7056 | Ling *et al*. (2013) |
| KD226945 | *Triticum urartu* cultivar G1812 unplaced genomic scaffold31185 | Ling *et al*. (2013) |
| KD191776 | *Triticum urartu* cultivar G1812 unplaced genomic scaffold6341 | Ling *et al*. (2013) |
| KD154067 | *Triticum urartu* cultivar G1812 unplaced genomic scaffold76628 | Ling *et al*. (2013) |
| KD250396 | *Triticum urartu* cultivar G1812 unplaced genomic scaffold4623 | Ling *et al*. (2013) |
| KD271846 | *Triticum urartu* cultivar G1812 unplaced genomic scaffold75656 | Ling *et al*. (2013) |
| KD039387 | *Triticum urartu* cultivar G1812 unplaced genomic scaffold81017 | Ling *et al*. (2013) |
| AY914082 | *Triticum monococcum* strain DV92 chromosome 5 clone 594O11 | Faris *et al*. (2003, 2008) |
| AY914083 | *Triticum monococcum* strain DV92 chromosome 5 clone 598P15-448N4 | Faris *et al*. (2003, 2008) |
| AY188331 | *Triticum monococcum* DV92 chromosome 5AL BAC 231A16 | Yan *et al*. (2003) |

**References**

Faris, J. D., J. P. Fellers, S. A. Brooks, B. S. Gill, 2003 A bacterial artificial chromosome contig spanning the major domestication locus *Q* in wheat and identification of a candidate gene. Genetics 164: 311-321.

Faris, J. D., Z. Zhang, J. P. Fellers, B. S. Gill, 2008 Micro-colinearity between rice, *Brachypodium*, and *Triticum monococcum* at the wheat domestication locus *Q*. Funct. Integr. Genomics 8: 149-164.

Gil-Humanes, J., F. Pistón, A. Martín, F. Barro, 2009 Comparative genomic analysis and expression of the *APETALA2-like* genes from barley, wheat, and barley-wheat amphiploids. BMC Plant Biol. 9: 66.

Jiang, Y. F., X. J. Lan, W. Luo, X. C. Kong, P. F. Qi, *et al*., 2014 Genome-wide quantitative trait locus mapping identifies multiple major loci for brittle rachis and threshability in Tibetan semi-wild wheat (*Triticum aestivum* ssp. *tibetanum* Shao). PloS one 9: e114066.

Kosuge, K., N. Watanabe, V. M. Melnik, L. I. Laikova, and N. P. Goncharov, 2012 New sources of compact spike morphology determined by the genes on the chromosome 5A in hexaploid wheat. Genet. Resour. Crop Evol. 59: 1115-1124.

Ling, H. Q., S. Zhao, D. Liu, J. Wang, H. Sun, *et al*., 2013 Draft genome of the wheat A-genome progenitor *Triticum urartu*. Nature 496: 87-90.

Yan, L, A. Loukoianov, G. Tranquilli, M. Helquera, T. Fahima, *et al*., 2003 Positional cloning of the wheat vernalization gene of *VRN1*. Proc. Natl. Acad. Sci., USA 100: 6253-6268.

Zhang, Z., H. Beclam, P. Gornicki, M. Charlesb, J. Justb, *et al*., 2011 Duplication and partitioning in evolution and function ofhomoeologous *Q* loci governing domestication characters in polyploid wheat. Proc. Natl. Acad. Sci., USA 108: 18737-18742.
